# Demographic Characteristics of Pneumoconiosis Cases: A Single Centre Experience

**DOI:** 10.2174/0115734056375744250225063355

**Published:** 2025-03-04

**Authors:** Bilge Akgündüz, Sermin Tok

**Affiliations:** 1 Occupational Diseases Clinic, Eskişehir City Hospital, 26080, Odunpazarı, Eskişehir, Türkiye; 2 Radiology Clinic, Eskişehir City Hospital, 26080, Odunpazarı, Eskişehir, Türkiye

**Keywords:** Pneumoconiosis, Profusion score, Pulmonary function, Smoking, Computed tomography, Interstitial lung diseases

## Abstract

**Background:**

Pneumoconiosis is a preventable occupational lung disease that is caused by the inhalation of inorganic occupational dust. The disease can progress and result in functional impairment. Profusion scores are crucial for the assessment of disease severity.

**Objective:**

This study aimed to determine the prevalence of pneumoconiosis cases with a profusion score of 0/1 and explore the correlation between pneumoconiosis and smoking behavior and sectors.

**Methods:**

A retrospective cross-sectional study was carried out in this work. Pneumoconiosis was diagnosed with occupational exposure histories and thoracic computed tomography (CT) findings. The study included patients admitted to the occupational diseases outpatient clinic at Eskişehir City Hospital for occupational or pulmonary conditions from January 2021 to July 2023. The collected data included age, sex, smoking status, pack-years, industry of employment, specific departments, occupations, exposure to occupational and non-occupational environmental factors, duration of exposure, laboratory results, pulmonary function test outcomes, thoracic CT findings, and International Classification of Radiographs of Pneumoconiosis score.

**Results:**

Among the 361 patients, 99.4% were male and 62.3% were current smokers. We observed a profusion score of 0/1 in 15% (n = 54) of the cases. Patients with a 0/1 profusion score had better lung function than those with higher scores, with the FEV1/FVC ratio declining as the profusion score increased. Non-smokers with progressive massive fibrosis had significantly lower FEV1/FVC ratios compared to other non-smokers.

**Conclusion:**

In order to avert the progression of early-stage cases, it is significant that we reevaluate occupational health policies and measures, regardless of compensation.

## 1 INTRODUCTION

Pneumoconiosis remains a significant occupational lung disease worldwide, with its prevalence and severity rising at alarming rates, posing serious threats to both public health and workplace safety. In 2021, pneumoconiosis caused by occu-pational particulate matter, gasses, and fumes resulted in approximately 4,775 fatalities and 117.80 thousand disability-adjusted life years [1]. Pneumoconiosis can progress even after the cessation of mineral dust exposure. Conventional screening methods often overlook a profusion score of 0/1, which indicates early-stage pneumoconiosis. Patients with a profusion score of 0/1 are frequently not recorded, even when a thoracic computed tomography (CT) scan is obtained, due to the reliance on X-ray radiography for compensation purposes [2]. Consequently, these individuals may continue working in environments where dust exposure persists.

Recent epidemiological studies have shown that minimal radiological findings classified as profusion score ≤ 0/1 can reflect significant dust exposure and early pathological changes. However, these early-stage cases are often under-represented in clinical trials and are excluded from occu-pational disease record-keeping systems, creating a gap between research and policy [3].

This study aimed to investigate the demographics, sectoral distribution, duration of exposure, smoking behaviors, and functional capacity of pneumoconiosis cases, including those with a profusion score of 0/1, and explore the correlation between disease severity and these factors. Additionally, it sought to address the cases with a profusion score of 0/1, which typically do not require occupational disease notification and are frequently overlooked in related studies [4].

## 2 MATERIALS AND METHODS

A retrospective cross-sectional study approach was utilized. Ethical approval was obtained from the Eskişehir Hospital ethics committee on August 17, 2023, under reference number ESH/GOEK-2023/44. The research study adhered to the ethical standards outlined in the Declaration of Helsinki. Due to the retrospective nature of the study, the IRB waived the requirement for informed consent. All methodologies were executed in accordance with relevant guidelines and regu-lations.

### 2.1 Study Population

This study included patients admitted to the occupational diseases outpatient clinic at Eskişehir City Hospital for occu-pational or pulmonary conditions from January 2021 to July 2023. The collected data included age, sex, smoking status, pack-years, industries of employment, specific departments, occupations, exposure to occupational and non-occupational environmental factors, duration of exposure, laboratory results, pulmonary function test outcomes, thoracic CT findings, International Labor Office (ILO) International Classification of Radiographs of Pneumoconiosis classification, and additional demographic variables. To rule out other interstitial lung diseases, additional testing, such as laboratory tests, bron-choalveolar lavage cytological and flow cytometric exami-nation, and tissue sample examinations, was carried out, as well as assessments of radiological findings by the Eskişehir City Hospital Chest Diseases and Pulmonary Occupational Disease Thoracic Radiology Council.

### 2.2 Inclusion Criteria

Participants were included if they were diagnosed with pneumoconiosis based on work history and thoracic CT abnormalities, defined according to the ILO International Classification of Radiographs of Pneumoconiosis with a profusion score of 0/1 or above. In order to rule out other lung diseases or interstitial lung diseases (ILD), cases requiring a differential diagnosis were subjected to additional testing, such as laboratory tests, tissue samples, cytological examinations, or assessments by the hospital's Chest Diseases and Pulmonary Occupational Disease Thoracic Radiology Council. The study included only individuals who had worked in the inorganic dust industry or divisions for a minimum of six months, as this was deemed a sufficient threshold for the identification of acute and accelerated silicosis cases.

### 2.3 Exclusion Criteria

Cases with missing data concerning characteristics, history of the working sector, occupation, working department, dura-tion of exposure, pulmonary function tests, smoking status, and diagnosis of other interstitial lung diseases (ILD), were excluded. Also, patients who had suboptimal CT findings, were under 18 years old, and had a duration of working time under 6 months, were excluded from the study.

### 2.4 Pulmonary Function Tests

High-precision devices (COSMED Quark PFT, Italy; reference no.: C03800-01-05, serial no.: 1015361935; COSMED Pony FX and COSMED Q-Box Body for desktop digital spirometry) were utilized to assess parameters, including forced vital capacity (FVC), forced expiratory volume in one second (FEV1), and FEV1/FVC ratios. These tests were used for the evaluation of functional loss and accom-panying airway obstruction in cases with pneumoconiosis. The tests were applied and interpreted according to ATS/ERS criteria. Airway obstruction was defined as the FEV1/FVC ratio being 70% or less in pulmonary function tests (PFTs) [5].

### 2.5 ILO International Classification of Pneumoconiosis Radiographs

Readings of quality 1 and quality 2 films were conducted. ILO classification assessments were conducted using lung X-rays obtained from our hospital's radiology department. Consequently, the pixel feature may be standardized for each lung X-ray.

Pneumoconiosis classes were determined according to the ILO pneumoconiosis classification guide. The patients' images were compared utilizing ILO DICOM standard films utilized universally. The classification was conducted independently by two physicians, and in the event of variations, a consensus had been reached.

Digital radiography was used to obtain chest X-rays (CXRs). CXRs were assessed following the ILO International Classification of Pneumoconiosis Radiographs. An occu-pational disease specialist, certified as an ILO International Pneumoconiosis Radiography Classification reader and responsible for occupational disease notification, conducted the evaluations. Small opacities were categorized into round and linear types, with profusion scores ranging from 0/- to 3+. Opacities larger than 1 cm were classified as A, B, or C. Profusion scores were categorized as 0, 1, 2, 3, and 4 [6].

#### 2.5.1 Profusion Scores

The term 'profusion of small opacities' describes the density of small opacities in the affected zones of the lung. The classification of profusion is determined by comparison with reference standard radiographs. While written descriptions provide guidance, the standard radiographs are the definitive basis for assessment. The increasing density of small opacities is evaluated accordingly [6].

The standard radiographs provide two examples of appea-rances classifiable as subcategory 0/0. Subcategory 0/0 refers to radiographs where there are no small opacities, or if a few are thought to be present, they are not sufficiently definite or numerous for category 1 to have been seriously considered as an alternative. Subcategory 0/1 is used for radiographs classified as category 0 after having seriously considered category 1 as an alternative. Subcategory 1/0 is used for radiographs classified as category 1 after having seriously considered category 0 as an alternative [6].

#### 2.5.2 Size

For shape and size, written descriptions served as a reference, but standard radiographs took precedence. The shape and size of small opacities were documented, with two types of shapes identified: rounded and irregular. Each shape was further classified into three size categories. For small rounded opacities, the sizes were labeled as p, q, and r, based on their appearance in the corresponding standard radiographs. These were defined as follows: p = opacities with diameters up to approximately 1.5 mm; q = opacities with diameters between 1.5 mm and 3 mm; r = opacities with diameters between 3 mm and 10 mm.

Similarly, the three size categories for small irregular opacities were labeled as s, t, and u, also defined by their appearance in the standard radiographs. These were specified as follows: s = opacities with widths up to about 1.5 mm; t = opacities with widths between 1.5 mm and 3 mm; u = opacities with widths between 3 mm and 10 mm [6].

#### 2.5.3 Large Opacities

A large opacity is defined as an opacity having the longest dimension exceeding 10 mm. The categories of large opacities are defined as follows: category A: one large opacity having the longest dimension up to about 50 mm, or several large opacities with the sum of their longest dimensions not exceeding about 50 mm; category B: one large opacity having the longest dimension exceeding 50 mm, but not exceeding the equivalent area of the right upper zone, or several large opacities with the sum of their longest dimensions exceeding 50 mm, but not exceeding the equivalent area of the right upper zone; category C: one large opacity exceeding the equivalent area of the right upper zone [6].

### 2.6 Cases Diagnosed with Pneumoconiosis

Cases that were evaluated with suspicion of pneumo-coniosis due to exposure to mineral dust underwent thorax CT, and after excluding other interstitial lung diseases with clinical, laboratory, radiological and, if necessary, cytological, patho-logical, and/or flow cytometric tests, all radiologic investi-gations were performed according to the ILO International Classification of Pneumoconiosis Radiographs by a radiologist and occupational diseases specialist.

### 2.7 Statistical Analysis

The Statistical Package for the Social Sciences for Windows 20.0 program was used for the statistical analysis of the data relevant to the cases (SPSS Inc.; Chicago, IL, USA). Frequencies and percentage values of categorical variables, and mean, median, and standard deviation values of numerical variables were calculated. Whether the variables showed normal distribution was tested with Skewness and Kurtosis measures. For numerical variables showing normal distri-bution, a t-test was applied in the presence of two groups, and an ANOVA test was applied in the presence of more than two groups. Posthoc Tukey HSD test was used to test a difference between the groups in numerical variables showing normal distribution. Categorical variables were tested with the chi-square test. Kruskal-Wallis test, which is a non-parametric test, was employed for variables with more than two groups that did not show normal distribution. Mann-Whitney U test was used to determine the difference within the group. Age, smoking status, cigarette pack-years, occupational exposure time, sector of employment, PFT results, ILO classification opacities in pneumoconiosis, pneumoconiosis profusion score category, and size were evaluated using an ordinal logistic regression test. The parallel lines test was used to verify that the assumption of multiple linearity of predictive variables was not violated and it was found to not be violated (p> 0.05). The significance result of the model was as follows: chi-square = 201.913, p < 0.001. This result showed the model to be statistically significant. Independent variables together explai-ned the dependent variable by approximately 46.4% (R2 = 0.464, Nagelkerke) (Table **S1**).

## 3 RESULTS

This study investigated 361 cases of pneumoconiosis documented at Eskişehir City Hospital, emphasizing the demo-graphic, clinical, and occupational characteristics of the patients. Among the patients, 99.4% (n: 359) were male and their average age was 46.3±7.9 years. The age range was 25-77 years. Smoking patterns exhibited variability, with 62.3% (n: 225) identified as current smokers, 15.2% as ex-smokers (n: 81), and 22.4% (n: 55) as never smokers, having an average of 20.2±11.9 pack years. The majority of patients [60.1% (n: 217)] had no symptoms, dyspnea was reported by 26.6% of the participants (n: 96), and coughing was present in 10.5% (n: 38) of the cases. The predominant comorbidities were chronic obstructive pulmonary disease (16%), tuberculosis (1%), and COVID-19 (29%) (Table **1a**).

**Fig. (1):**
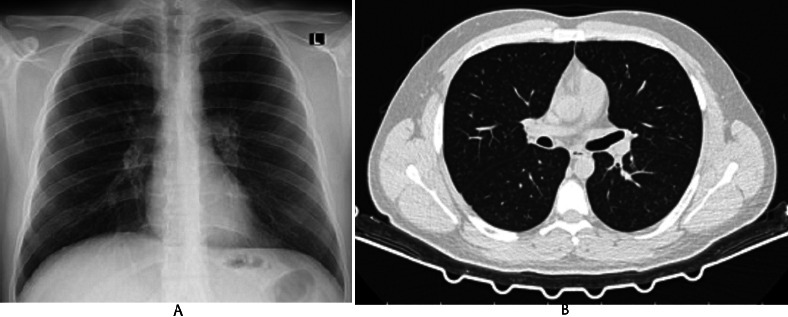
**A**: ILO pneumoconiosis PA lung X-ray classification p/p 1/1. **B**: Lung CT with small opacities. **Note: **This radiological image originates from a patient followed by the author in clinical practice in occupational diseases clinic.

**Fig. (2):**
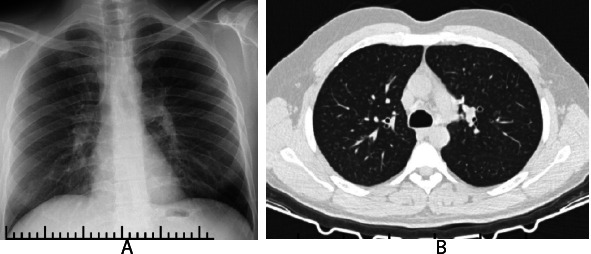
**A**: ILO pneumoconiosis PA lung X-ray classification p/p 0/1. **B**: Lung CT with small opacities. **Note: **This radiological image originates from a patient followed by the author in clinical practice in occupational diseases clinic.

**Fig. (3):**
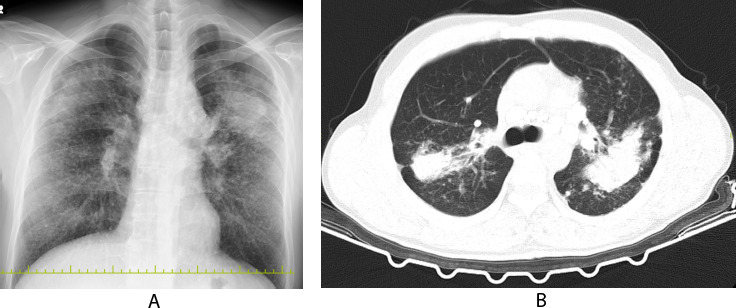
**A**: ILO pneumoconiosis PA lung X-ray classification q/q 3/3 + B opacity. **B**: Lung CT with small opacities and large opacity. **Note: **This radiological image originates from a patient followed by the author in clinical practice in occupational diseases clinic.

Approximately 57% of the subjects (n: 206) were emp-loyed in the ceramic industry, and the average duration of exposure to inorganic dust was 17.4±8.4 years. 8.1% of the cases (n: 29) had a history of working in the glass industry, 1.7% (n: 6) in cat litter manufacturing, and 3 cases had a history of working in the plastic industry with thermoplastic polymer exposure. 50.7% of the cases (n: 183) had a profusion score category of 1 (Fig. **1**). In total, 15% (n: 54) of the cases had a profusion score of 0/1 (Fig. **2**). Large opacities were detected in 8.3% (n: 30) of the cases (Table **1b**; Fig. **3**).

Pulmonary function tests showed an average FVC of 98.2% ± 21.5, FEV1 of 95.3% ± 23.1, and a FEV1/FVC ratio of 79.0% ± 8.8. PFTs were elevated among non-smokers compared to smokers and severe disease (category 4) has shown a notable decrease in the FEV1/FVC ratio among smokers (71.33%, p = 0.001). A clear correlation existed between exposure and response; lower profusion scores (e.g., 0/1) were associated with shorter exposure periods (14.14 ± 7.8 years), whereas higher categories were associated with longer exposure durations (up to 19.5 ± 7.8 years) (Tables **2** and **3**). In cases with larger opacity sizes and higher profusion scores, the duration of exposure was generally prolonged (Table **3**).

The evaluation of the pneumoconiosis category (stage), according to smoking status, cigarette pack-years, occupational exposure time, working sector, pulmonary function test results, and sizes of opacities, was carried out by using the ordinal logistic regression test. The parallel lines test was used to verify that the multicollinearity assumption of the predictor variables was not violated and it was found to not be violated (p> 0.05). A significant relationship was found between the opacity size and the FEV1/FVC ratio (Wald statistic = 6.931, P = 0.008). The odds ratio was found to be 0.911 (95% CI = 0.850-0.977). It was seen that increasing the opacity size by one unit reduced the FEV1/FVC ratio by approximately 0.9 times. A significant relationship was also found between the degree of opacity size and opacity profusion (Wald statistic = 80.591, P<0.001). The odds ratio was found to be = 9.116 (95% CI = 5.626-14.769). It was also observed that increasing the opacity size by one unit increased the pneumoconiosis stage by approximately 9 times. Among the categorical variables, with reference to the sector variable, there were significant correlations found between the dusty sectors other than the ceramics, such as cat litter, marble, construction, and thermoplastic factories, as well as metal and mineral glass sectors (variables coded as ceramics: Wald statistic = 7.671, P=0.006; the variable coded as metal: Wald statistic = 6.232, P=0.013; the variable coded as glass: Wald statistic = 6.758, P = 0.009; and the variable coded as mining: Wald statistic = 5.971, P = 0.015). The odds ratios and 95% confidence intervals were 987.352 (7.506 - 129872.845) for ceramics, 517.092 (3.829 - 69838.018) for metal, 780.610 (5.149 - 118352.916) for glass, and 523.351 (3.451 - 79361.143) for mineral, respectively. In other words, we can say that ceramics posed approximately 987 times more risk, metal 517 times more risk, glass 780 times more risk, and mining 523 times more risk than professions coded as others (Table **4**).

## 4 DISCUSSION

### 4.1 Profusion Score 0/1 in Small Opacities

Using logistic regression models, we demonstrated the profusion score to increase exponentially as the opacity size increased in our study. The significance of exposure time in the progression of pneumoconiosis in occupations associated with dust exposure was demonstrated by the substantial correlation among opacity size, profusion score, and working hours. The literature contains a limited number of studies on pneumoconiosis with a profusion score of 0/1 or lower. The agreement between ILO pneumoconiosis readers or the relationship between ILO classification and thoracic CT findings has been the primary focus of studies including category 0 [7-10]. The majority of pneumoconiosis studies involve profusion scores of 1/10 or higher. Nevertheless, pneumoconiosis necessitates the consideration of a profusion score of 0/1. Our study determined the rate of 0/1 profusion in pneumoconiosis to be 15%. Miller et al. found that 19.76% of individuals exposed to asbestos had a profusion score of 0/1 [11]. In another study conducted in various sectors of Turkey, small opacity profusion was reported to be 0/1 in 25% of pneumoconiosis cases [12]. Although Blanc and other researchers suggested that omitting the 0/1 score could introduce bias, the data demonstrated a 0/1 profusion score at a substantial frequency in pneumoconiosis cases [13].

Consequently, clinicians should implement appropriate measures to diagnose the disease at an early stage and stop its progression. Our research study demonstrated that the profusion score increased by up to 48 times as the extent of the opacity increased. Studies define pneumoconiosis as having an ILO pneumoconiosis profusion score of 1/0 or higher, excluding scores of 0/1 or lower. Welder's pneumoconiosis is the most paradigmatic type of category 0 pneumoconiosis [14]. Although chest radiography is within the normal range in Welder's pneumoconiosis, thoracic CT findings are characterized by widespread micronodules with inadequately defined centrinodular ground glass density in the upper lobes [15]. In the study conducted by Takahashi et al., round opacities were detected in 37 of 66 cases with Welder’s pneumoconiosis in tomography, while irregular opacities were detected in 57. The profusion score of the cases was assessed as 0 [16]. Category 0 is defined as minor opacities that are absent or less profuse than category 1 on standard radiographs in the ILO pneumoconiosis classification [6]. It is inappropriate to define pneumoconiosis as category 1 and above, as per the guideline. However, pneumoconioses are defined as parenchymal lung diseases that result from the inhalation of inorganic dust in the workplace [7]. It is evident that the profusion score or category is not used in the diagnosis of pneumoconiosis; these definitions are terms that are utilized in pneumoconiosis screening and follow-up and are associated with insurance compensation. The National Institute for Occupational Safety and Health has stated that parenchymal abnormalities, particularly small opacities with profusion classifications of 1/0 or greater, are frequently regarded as compatible with pneumoconiosis in compensation cases [17, 18]. In the fight against pneumoconiosis, the exclusion of 0/- and 0/1 cases from investigations may lead to incomplete rates of prevalence and incidence.

### 4.2 Pneumoconiosis According to Sectors and its Effect on ILO Classification

The ceramic sector's pneumoconiosis incidence was found to be high during the assessment of pneumoconiosis studies in Turkey [12, 19]. It was also corroborated by our research. Moreover, our investigation encompassed pneumoconiosis cases that were employed in cat litter plants and the glass industry. There is a lack of precise data regarding the incidence of pneumoconiosis in the glass sector, despite the fact that several studies in the literature have suggested silica exposure to be high [20-22]. This study is the first to suggest that the glass industry has a substantial number of pneumoconiosis cases. Silica exposure should not be disregarded in the production of cat litter that contains bentonite [23]. Once more, this investigation is the first to conduct a comprehensive analysis of pneumoconiosis cases on a sectoral scale in the production of cat litter. Our research study has demonstrated the ceramic, metal, glass, and mining sectors to be at risk due to opacity size, and that a 1-unit increase in profusion score results in a 48-fold increase in size. It is feasible to assert that pneumoconiosis becomes simpler to identify as the diameter of the opacity increases. The diagnosis may be more challenging with standard chest radiography, particularly in cases where opacities are less than 1.5 mm and profusion scores are 0/1 or 1/0. Failure to diagnose the disease during the early onset stage or continued exposure may result in the failure to take preventative measures, which could potentially lead to pneumoconiosis outbreaks in these sectors in the future.

The duration of exposure and the time it takes for pneumo-coniosis to develop are subject to variation in the literature [23-25]. The average exposure duration of cases developing pneumoconiosis was 17.4 years in our study. The exposure duration of cases with a profusion score of 0/1 was determined to be shorter than that of cases with a profusion score of 1/0 and higher. Profusion scores were found to be higher in dental technicians who had a longer exposure duration in a study [26]. Once more, a meta-analysis that encompassed 19 studies determined the duration of dust exposure to be a risk factor for the development of pneumoconiosis [4]. This research also suggests that the disease can be detected in a more visible stage, specifically the fibrosis stage, if the dust exposure persists.

### 4.3 Smoking Behaviour and the Effect of Pneumoconiosis on Pulmonary Function Tests

The terms “smoker's lung” and “dirty chest” are employed in lung radiography to describe the condition of smokers. Pneumoconiosis screening chest X-ray findings may be mistaken for smoker's lung [27, 28]. In respiratory bronchiolitis associated with smoking and in the early onset of pneumo-coniosis, ground-glass density micronodules with indistinct contours can be observed on thoracic CT in the upper lobes [27, 28]. This may be a confounding factor that influences the relationship between silicosis and smoking. In the review conducted by Hessel et al. regarding the correlation between smoking and silicosis, it was noted that a biological gradient could not be evaluated between the severity of silicosis and smoking due to the fact that the majority of studies included categories with a threshold of ≥1/0 [29]. It is hypothesized that the findings of certain studies may be influenced by the incorrect classification of radiographic changes caused by smoking as pneumoconiosis. In this investigation, no correlation was observed between smoking behavior and profusion score categories. Simultaneously, the study cohort comprised cases in which pneumoconiosis was diagnosed by conducting a differential diagnosis of pneumoconiosis from other interstitial lung diseases, followed by additional examination as required. In this regard, it can be concluded that the study effectively mitigated confounding factors. There has been a long-standing understanding of the impact of smoking on airway obstruction [30, 31]. The number of studies that have investigated the impact of smoking behaviour on pulmonary function tests in individuals with pneumoconiosis is quite limited. It has been reported that the FEV1/FVC ratio is low in individuals with pneumoconiosis [32]. The FEV1/FVC and FEV1 ratios of non-smoking individuals with pneumoconiosis were found to be lower than those with a history of smoking in our study. This is an intriguing finding. Furthermore, the FEV1/FVC ratio of non-smoking category 4 cases, which are classified as having large opacities, was determined to be significantly lower than that of category 1 cases with a profusion score of 0/1. This implies that the progression of the disease may result in airway obstruction and a loss of pulmonary function due to inflammation caused by mineral particles. This is a hypothetical assertion that necessitates additional research.

### 4.4 Bias and Limitations

Our study recognized a pronounced gender disparity, comprising 359 male individuals and merely 2 female participants. This skewed distribution reflects the actual occupational demographics of the industry where the study has been done, rather than a sampling bias. The industry in issue has a largely male workforce, resulting in a significant overrepresentation of male participants in our sample. Due to the significant gender imbalance, the application of statistical re-weighting procedures or the execution of separate gender-specific studies proved impractical. The limited number of female cases (n=2) may not yield statistically significant comparisons or dependable subgroup analyses. We openly acknowledge this limitation and stress that our findings mostly represent the impacts of exposure on male workers.

The region our hospital serves is the region with the highest number of ceramic factories in Turkey. Although selection bias is mentioned in this regard, it also points to the prevalence of pneumoconiosis in the ceramic industry in Turkey.

This investigation has been confronted with numerous limitations. Initially, the retrospective nature of the study made it challenging to ascertain the cause-and-effect relationship between the severity of pneumoconiosis and exposure levels. Secondly, diagnostic methods, such as chest radiographs and CT scans, may not have identified early signs or necessitated subjective interpretation by radiologists. Third, the genera-lizability of the findings may be restricted by the fact that the patients of a single hospital may not be representative of all those exposed. Fourth, the accuracy levels may be impacted by the introduction of recall bias, which could result from relying on self-reported data for occupational exposure. In order to enhance the reliability and applicability of the data, it is recommended that more objective measures be implemented in conjunction with a broader participation base in the future.

Our hospital is the only hospital in the nearby region with an occupational diseases polyclinic. Therefore, it is the hospital to which workplace physicians and branch physicians are referred not only from Eskişehir, but also from surrounding cities in case of suspicion of occupational disease. Although our study involved a single-center experience, the results may reflect the findings of the whole region.

## CONCLUSION

This study emphasized two critical aspects to consider when dealing with silicosis cases: first, early diagnosis, particularly in those with profusion scores of 0/1, and second, respiratory function decline in advanced disease even in the absence of smoking. Our findings have indicated frequently subtle radiological signs that may be undetectable by standard imaging, but may indicate major pathological alterations in the early stages. This study on occupational lung illness has offered insights into the risk variables and prediction modeling of pneumoconiosis in a practical occupational context. Considering the substantial effect of pneumoconiosis on occupational health, our results have emphasized risk assessment methodologies and early detection initiatives. This research underscores the importance of customized preventive strategies, especially for high-risk occupational categories. Subsequent research ought to prioritize the integration of larger and more equitable datasets to enhance model generalizability. Furthermore, investigating sophisticated machine learning models, including ensemble learning, deep learning methodo-logies, and explainable AI approaches, may augment predicted efficacy and interpretability. Mitigating gender disparity and assessing potential sex-related variations in illness develop-ment using more inclusive datasets may prove to be essential in future research. These findings underline the importance of enhanced screening techniques in high-risk industries in order to prevent such diseases from progressing to more severe stages. Finally, proactive efforts to stop occupational exposure are required to avoid the spread of pneumoconiosis among workers.

## Figures and Tables

**Table:** 

Categories	Category 0			Category 1			Category 2			Category 3			Category 4
Subcategories	0/-	0/0	0/1	1/0	1/1	½	2/1	2/2	2/3	3/2	3/3	3/+	Large opacity

**Table 1a:** Descriptive characteristics of 361 patients.

**Descriptive Characteristics**	-	**N**	**%**
**Age, year, mean ±SD**	46.3 ± 7.9	-	-
**Gender**
	Male	359	99.4
	Female	2	0.6
**Smoking status**
	Current smoker	225	62.3
	Ex-smoker	55	15.2
	Non-smoker	81	22.4
**Smoking pack years, mean ±SD**	20.2 ± 11.9	-	-
**Symptoms**
	None	217	60.1
	Cough	38	10.5
	Dyspnea	96	26.6
	Cough and dyspnea	10	2.8
**Sector**
	Ceramic	206	57.1
	Foundry	36	10.0
	Glass	29	8.1
	Welding	24	6.7
	Metal	17	4.7
	Mining	17	4.7
	Brick and tile	9	2.5
	Cement	7	1.9
	Cat litter	6	1.7
	Marble	4	0.8
	Construction	3	0.8
	Thermoplastic and PVC	3	0.8
**Duration of exposure, year, mean ±SD**	17.4 ± 8.2	-	-
**History of pulmonary disease**
	Tuberculosis	7	1.9
	Bronchiectasis	3	0.08
	COPD	59	16.4
	Asthma	12	0.02
	Pneumonia	18	5.0
	COVID-19 history	107	29.5
	Environmental asbestos-related pleural thickening (plaques) and calcification	4	1.1
	Emphysema	7	1.9
**Pet bird keeping and pigeon keeping history**	-	88	24.4
**Moisture and mold at home history**		21	5.8
**Present musculoskeletal disease**		64	17.7
**Work accident history**		89	24.5
**Pulmonary function test**		-	-
**FVC, mL**	4.35 ± 1.06	-	-
**FVC, %**	98.2 ± 21.5	-	-
**FEV1, mL**	3.45 ± 0.95	-	-
**FEV1, %**	95.3 ± 23.1	-	-
**FEV1/FVC**	79.0 ± 8.8	-	-
**Diffusion test**	-		
**DLCO, %**	89.9±24.2	-	-
**DLCO/VA, %**	92.7±18.8	-	-
**TLC, %**	94.6±20.6	-	-

**Abbreviations:** COPD: Chronic obstructive pulmonary disease; FVC: Forced vital capacity; FEV1: Forced expiratory volume exhaled in the first second; DLCO: Diffusion capacity; DLCO/VA: Diffusion capacity divided by the alveolar volüme; TLC: Total lung capacity.

**Table 1b:** ILO classification parameters (n=361 patients).

**ILO classification**		**N**	**%**
**Profusion score category**	0	54	15.0
	1	183	50.7
	2	82	22.7
	3	12	3.3
	4	30	8.3
	Total	361	100
**Small opacity size**	<1.5 mm	287	79.5
	1.5-3 mm	65	18.0
	>3	9	2.5
	Total	361	100
**Large opacity**	A	17	4.7
	B	3	.8
	C	10	2.8
	Total	30	8.3

**Table 2:** Comparison of categories according to smoking behaviour in cases with pneumoconiosis (n = 361 patients).

**Smoking behaviour**	**PFT**	**ILO Classification Category**	**N**	**Mean**	**Std. deviation**	**F-ratio**	**p**	**LSD**
**Ever**	**FVC, %**	**0**	47	94.70	21.65	1.929	0.106	-
		**1**	161	100.32	19.85			-
		**2**	69	96.61	20.72			-
		**3**	7	94.00	26.07			-
		**4**	22	89.36	19.79			-
		**Total**	306	97.69	20.59	-	-	-
	**FEV1, %**	**0**	47	92.26	19.57	3.489	0.008	-
		**1**	161	97.56	21.08			(1,4)
		**2**	69	94.86	22.63			(2,4)
		**3**	7	90.29	26.70			-
		**4**	22	80.00	24.03			(4,1),(4,2)
		**Total**	306	94.71	21.91	-	-	-
	**FEV1/FVC**	**0**	47	77.71	9.58	5.283	0.001	(0,4)
		**1**	161	79.89	7.89			(1,4)
		**2**	69	79.36	8.29			(2,4)
		**3**	7	80.40	6.02			-
		**4**	22	71.33	10.93			(4,0),(4,1),(4,2)
		**Total**	306	78.84	8.70	-	-	-
**Never**	**FVC, %**	**0**	7	103.27	22.76	.458	0.766	-
		**1**	22	105.95	27.95			-
		**2**	13	99.08	23.73			-
		**3**	5	97.40	30.25			-
		**4**	8	92.25	28.02			-
		**Total**	55	101.23	26.081	-	-	-
	**FEV1, %**	**0**	7	107.00	22.51	1.809	0.142	-
		**1**	22	106.23	27.84			-
		**2**	13	97.54	24.75			-
		**3**	5	95.60	30.78			-
		**4**	8	76.88	34.28			-
		**Total**	55	99.04	28.64	-	-	-
	**FEV1/FVC**	**0**	7	86.87	4.76	4.869	0.002	(0,4)
		**1**	22	82.25	4.59			(1,4)
		**2**	13	79.92	5.31			-
		**3**	5	78.24	7.44			-
		**4**	8	69.31	18.45			(4,0),(4,1)
		**Total**	**55**	**80.04**	**9.52**	-	-	-

**Abbreviations**: PFT: Pulmonary function test; FVC: Forced vital capacity; FEV1: Forced expiratory volume exhaled in the first second; DLCO: Diffusion capacity; DLCO/VA: Diffusion capacity divided by the alveolar volüme; TLC: Total lung capacity; LSD: Least significant difference.

**Table 3:** Comparison of the duration of inorganic dust exposure by pneumoconiosis classifications according to the categories and small opacity sizes.

**ILO Classification**		**N**	**Mean**	**Std. deviation**	**F**	**p**	**LSD**
**Category**	0	54	14.14	7.87	3.956	0.004	(0,2),(0,4)
	1	183	17.06	8.41	-		-
	2	82	18.97	7.82	-		(2,0)
	3	12	20.41	6.10	-		-
	4	30	19.50	7.85	-		(4,0)
	Total	361	17.37	8.22	-		-
**Small opacity size**	<1.5 mm	233	17.42	8.46	3.261	0.040	(<1.5 vs. 1.5-3)
	1.5-3 mm	65	20.14	6.54	-		-
	>3	9	15.56	9.01	-		(1.5-3 vs. <1.5)
	Total	307	17.94	8.17	-	-	-

**Abbreviations:** LSD: Least Signi̇fi̇cant Di̇fference.

**Table 4:** Ordinal logistic regression analysis on pneumoconiosis opacity sizes related to the duration of exposure, sectors, and health variables.

-	**Variable**	**Estimate**	**Std. error**	**Wald**	**df**	**Sig.**	**95% Confidence Interval**		**Odds Ratio (OR)**	**95% Confidence Interval**	
							**Lower Bound**	**Upper Bound**		**Lower Bound**	**Upper Bound**
Threshold	Opacity size (<1,5 mm)	2.834	3.564	0.632	1	0.427	-4.151	9.820	-	-	-
	Opacity size (1.5-3 mm)	6.724	3.612	3.466	1	0.063	-0.355	13.803	-	-	-
Location	Duration of exposure, year	0.724	0.454	2.541	1	0.111	-0.166	1.615	2.063	0.847	5.026
	Smoking pack years	0.008	0.015	0.283	1	0.595	-0.022	0.038	1.008	0.979	1.038
	FVC %	-0.005	0.023	0.048	1	0.827	-0.051	0.041	0.995	0.950	1.042
	FEV1%	-0.006	0.025	0.062	1	0.803	-0.054	0.042	0.994	0.947	1.043
	FEV1/FVC %	-0.093	0.035	6.931	1	0.008	-0.162	-0.024	0.911	0.850	0.977
	Ceramic	6.895	2.489	7.671	1	0.006	2.016	11.774	987.352	7.506	129872.845
	Metal	6.248	2.503	6.232	1	0.013	1.343	11.154	517.092	3.829	69838.018
	Glass	2.667	2.623	1.034	1	0.309	-2.474	7.809	14.402	0.084	2462.052
	Mining	6.660	2.562	6.758	1	0.009	1.639	11.681	780.610	5.149	118352.916
	Brick and cement	6.260	2.562	5.971	1	0.015	1.239	11.282	523.351	3.451	79361.143
	Ever smoker	-0.908	0.514	3.126	1	0.077	-1.915	0.099	0.403	0.147	1.104
	Present FEV1/FVC< 70%	-1.158	0.669	2.993	1	0.084	-2.470	0.154	0.314	0.085	1.166

**Abbreviations:** FVC: Forced vital capacity; FEV1: Forced expiratory volume exhaled in the first second **Note:** Odds ratios (OR) and 95% confidence intervals provide a measure of the association strength and reliability. The model’s overall significance and model fit indices (Chi-square, Nagelkerke R^2^) have been summarized at the end to confirm the robustness of the analyses.

## Data Availability

The data of current study are available from corresponding author [B.A], on a reasonable request.

## LIST OF ABBREVIATIONS

CT: Computed tomography
ILO: International Labor Office
ILD: Interstitial lung diseases
FVC: Forced vital capacity
FEV1: Forced expiratory volume in one second
PFTs: Pulmonary function tests
CXRs: Chest X-rays

## STANDARDS OF REPORTING

STROBE guidelines were followed.
