# Supplementary material for: Demographic Characteristics of Pneumoconiosis Cases: A Single Centre Experience
Source: Curr Med Imaging. 2025 Mar 4;21:e15734056375744. doi: 10.2174/0115734056375744250225063355 (PMC13096873; doi:10.2174/0115734056375744250225063355)
Supplement: Supplementary file 1 [file CMIM-21-E15734056375744_SD1.pdf]

1573-4056/25

Send Orders for Reprints to reprints@benthamscience.net

BENTHAM SCIENCE

CrossMark

Current Medical Imaging

Content list available at: https://benthamscience.com/journals/cmir

Demographic Characteristics of Pneumoconiosis Cases: A Single Centre Experience

Bilge Akgündüz[1,*] and Sermin Tok[2]

[1]*Occupational Diseases Clinic, Eskişehir City Hospital, 26080, Odunpazarı, Eskişehir, Türkiye*
[2]*Radiology Clinic, Eskişehir City Hospital, 26080, Odunpazarı, Eskişehir, Türkiye*

Article History	Received: December 23, 2024	Revised: February 09, 2025	Accepted: February 19, 2025

Table S1. Ordinal logistic regression analysis on pneumoconiosis categories related to duration of exposure, sectors and health variables.

	Variable	Estimate	Std. Error	Wald	df	Sig.	95% Confidence Interval		Odds Ratio (OR)	95% Confidence Interval	
							Lower Bound	Upper Bound		Lower Bound	Upper Bound
Threshold	Category 0	2.983	2.163	1.902	1	0.168	-1.257	7.223			
	Category 1	6.030	2.171	7.716	1	0.005	1.775	10.285			
	Category 2	8.554	2.221	14.836	1	0.000	4.201	12.906			
	Category 3	9.171	2.231	16.892	1	0.000	4.797	13.544			
Location	Duration of exposure, year	-0.001	0.250	0.000	1	0.997	-0.491	0.489	0.999	0.612	1.631
	Smoking pack years	0.002	0.010	0.054	1	0.816	-0.017	0.022	1.002	0.983	1.022
	FVC %	0.026	0.016	2.497	1	0.114	-0.006	0.058	1.026	0.994	1.059
	FEV1%	-0.027	0.017	2.671	1	0.102	-0.060	0.005	0.973	0.941	1.005
	FEV1/FVC %	0.015	0.023	0.428	1	0.513	-0.029	0.059	1.015	0.971	1.061
	Opacity size (1.5-3 mm)	3.877	0.391	98.554	1	0.000	3.112	4.643	48.286	22.458	103.816
	Ceramic	-0.228	0.825	0.077	1	0.782	-1.846	1.389	0.796	0.158	4.012
	Metal	-0.515	0.842	0.374	1	0.541	-2.165	1.135	0.597	0.115	3.111
	Glass	-0.527	0.901	0.342	1	0.559	-2.293	1.240	0.591	0.101	3.455
	Mining	-0.777	0.901	0.743	1	0.389	-2.544	0.990	0.460	0.079	2.690
	Brick and Cement	-0.420	0.944	0.198	1	0.656	-2.271	1.431	0.657	0.103	4.182
	Ever smoker	-0.235	0.364	0.417	1	0.518	-0.948	0.478	0.791	0.388	1.613
	Present FEV1/FVC< 70%	-1.158	0.669	2.993	1	0.084	-2.470	0.154	0.314	0.085	1.166

Abbreviations: FVC: Forced vital capacity; FEV1: Forced expiratory volume exhaled in the first second **Notes:** Odds Ratios (OR) and 95% Confidence Intervals provide a measure of the association strength and reliability. The model's overall significance and model fit indices (Chi-square, Nagelkerke R^2) have been summarized at the end to confirm the robustness of the analyses.

© 2025 The Author(s). Published by Bentham Science Publisher.

This is an open access article distributed under the terms of the Creative Commons Attribution 4.0 International Public License (CC-BY 4.0), a copy of which is available at: https://creativecommons.org/licenses/by/4.0/legalcode. This license permits unrestricted use, distribution, and reproduction in any medium, provided the original author and source are credited.

DOI: 10.2174/0115734056375744250225063355, 2025, *21*, e15734056375744
